# Alpha 5 Integrin Mediates Osteoarthritic Changes in Mouse Knee Joints

**DOI:** 10.1371/journal.pone.0156783

**Published:** 2016-06-09

**Authors:** Maria Elena Candela, Chao Wang, Aruni T. Gunawardena, Kairui Zhang, Leslie Cantley, Rika Yasuhara, Yu Usami, Noelle Francois, Masahiro Iwamoto, Arjan van der Flier, Yejia Zhang, Ling Qin, Lin Han, Motomi Enomoto-Iwamoto

**Affiliations:** 1 Department of Surgery, Division of Orthopaedic Surgery, Children’s Hospital of Philadelphia, Philadelphia, PA, United States of America; 2 School of Biomedical Engineering Science, and Health Systems, Drexel University, Philadelphia, PA, United States of America; 3 Department of Orthopaedics and Traumatology, Nanfang Hospital, Southern Medical University, Guangzhou, China; 4 Division of Pathology, Department of Oral Diagnosis Science, School of Dentistry, Showa University, Tokyo, Japan; 5 Howard Hughes Medical Institute, Koch Institute for Integrative Cancer Research, Massachusetts Institute of Technology, Cambridge, MA, United States of America; 6 Department of Physical Medicine and Rehabilitation, Perelman School of Medicine, University of Pennsylvania, Philadelphia, PA, United States of America; 7 Department of Orthopaedic Surgery, University of Pennsylvania, Philadelphia, PA, United States of America; Van Andel Institute, UNITED STATES

## Abstract

Osteoarthritis (OA) is one of most common skeletal disorders and can affect synovial joints such as knee and ankle joints. α5 integrin, a major fibronectin receptor, is expressed in articular cartilage and has been demonstrated to play roles in synovial joint development and in the regulation of chondrocyte survival and matrix degradation in articular cartilage. We hypothesized that α5 integrin signaling is involved in pathogenesis of OA. To test this, we generated compound mice that conditionally ablate α5 integrin in the synovial joints using the *Gdf5Cre* system. The compound mice were born normally and had an overall appearance similar to the control mice. However, when the mutant mice received the OA surgery, they showed stronger resistance to osteoarthritic changes than the control. Specifically the mutant knee joints presented lower levels of cartilage matrix and structure loss and synovial changes and showed stronger biomechanical properties than the control knee joints. These findings indicate that α5 integrin may not be essential for synovial joint development but play a causative role in induction of osteoarthritic changes.

## Introduction

Osteoarthritis (OA) is one of the most common skeletal diseases and involves pathological changes in synovial joint components such as articular cartilage and synovium. Pathological changes seen in articular cartilage in OA include irregularity and loss of articular surface, loss of proteoglycan matrix and alterations of collagen fibrils and fibers, and cleft and erosion of articular cartilage [1]. These changes result from many cellular events including cytoskeletal changes, proliferation, matrix synthesis and degradation, cell senescence and apoptosis, as well as hypertrophy [2]. In addition to alterations of cartilage structure and function, thickening of subchondral bone and ectopic bone formation (osteophyte) occur in OA [3, 4]. Synovial changes, including synovitis and hyperplasia of synovial cells, are found in synovial joints from the early stages of osteoarthritis, and have been studied as causative events and therapeutic targets of this condition [5, 6]. Continuous and extensive efforts have been made to understand the biology of joint homeostasis and the pathogenesis of OA for the development of therapy for this disease, though no effective disease-modifying osteoarthritis drug has been discovered.

Alpha 5 integrin (α5 integrin) is a fibronectin receptor that mediates a variety of biological phenomena including mesoderm induction, vascular development, and neural crest development [7–10]. Human articular chondrocytes express α5β1 together with many other heterodimers of integrins such as α1β1, αvβ5, αvβ3 and α3β1 [11, 12]. Among these integrins, α5β1 has been demonstrated to play roles in synovial joint development [13] and in the regulation of chondrocyte survival, matrix degradation, and expression of cytokines and non-cartilaginous collagens [14–18]. Based on these findings, we hypothesized that α5 integrin signaling is involved in pathogenesis of OA. To test this hypothesis, we generated conditional knockout mice lacking α5 integrin expression specifically in synovial joints through use of the GDF5Cre system [19, 20] and analyzed the pathological and biomechanical changes of knee joints after OA surgery. The findings suggest that α5 integrin mediates the progression of OA, possibly targeting synovial cells.

## Materials and Methods

### Mice

All animal experiment procedures were approved by the Institutional Animal Care and Use Committee of the Children’s Hospital of Philadelphia. The Institute maintains veterinary personnel who supervise our work and answer any question we may have related to the use and care of vertebrate animals. When pain/distress are observed, the animals will be treated with Buprenex (0.1–2.0 mg/kg), as well as crushed or wet food. If pain or distress continues, we will euthanize the mouse regardless of the scheduled endpoints. The criteria that determine discomfort/distress/pain are any three of the following signs: Abnormal posture, slow, careful or abnormal (waddling) gait, low activity, slow eating, cowering or vocalizing while handling, change in eye or coat appearance and weight loss. We strictly follow the American Veterinary Medical Association Panel on Euthanasia for adult mice and NIH Euthanasia Guidelines for mouse embryos and neonates. Specifically, mice older than 14 days will be euthanized by CO_2_ inhalation. We will assure euthanasia by one of the following criteria: Cervical dislocation after no response to tail or toe pinch, no respiration or heartbeat after thirty seconds continuous monitoring, or rigor mortis. We will give anesthesia (isoflurane inhalation) to mouse embryos older than E15 and neonates up to 14 days prior to decapitation. Mice will be anesthetized by inhalation of 1–5% of isoflurane during OA surgery and tail clipping for genotyping.

α5 integrin was conditionally knocked out of the synovial joints by generation of the triple compound transgenic mouse that encodes *Cre* under the control *Gdf5* expression (*Gdf5Cre*) [19], *flox* α*5 integrin* [9] and α*5 null* alleles [8]. First we mated the *Gdf5Cre* mouse with the α*5 null* mouse. The resulting double-het (*Gdf5Cre;* α*5null/wt*) mouse was mated with homozygous floxed α5 integrin mouse *(*α*5fl/fl*) to generate the triple compound mouse (*Gdf5Cre;* α*5null/fl*, hereafter called α5 CKO). The genotyping for the *Gdf5Cre*, α*5 null* and *flox* α*5* alleles and the excised allele of the *floxed* α*5* have been described elsewhere [9]. The *Gdf5Cre;* α*5fl/wt*, α*5fl/null* or α*5fl/wt* were used as the control since they do not show significant differences in OA phenotype after OA surgery. To monitor the fate of Gdf5-lineage cells, we generated the Gdf5Cre;Rosa-GFP compound mouse. To determine localization of α5 integrin, we used C57BL/6j (Jackson Lab, Bar Harbor, ME, USA) mice.

### OA surgery

Under anesthesia, we transected the medial collateral ligament and removed the medial meniscus in the right knee in both α5 CKO and control littermate male mice (n = 9/group) at 3 months of age as described previously [21]. Knees were collected and examined by histological and histochemical means 1 month after surgery.

### Histology and immunostaining

The knee joints were fixed with 10% formalin or 4% paraformaldehyde overnight and decalcified with EDTA for 2 weeks. The paraffin sections were stained with hematoxylin and eosin or Safranin O staining. To detect apoptotic cells, we performed TUNEL staining using the ApopTag peroxidase *in situ* apoptosis detection kit (EMD Millipore, Billerica, MA, USA) and the In Situ Cell Death Detection Kit, Fluorescein (Roche, Branford, CT, USA). For immunohistochemical staining for collagen 10, MMP13, VDIPEN, CD31, and GFP, the paraffin sections were pre-treated with 0.1% pepsin (collagen 10 and MMP13: 10 min at 37°C) and/or 10 mM sodium citrate (MMP13, VDIPEN, CD31 and GFP: 10 min at 95°C) and then incubated with primary antibodies (collagen 10: Cosmo Bio USA (Carisbad, CA, USA), 1:1000; MMP13: Abcam (Cambidges, UK), 1:250; VDIPEN (gift from Dr. J. Mort (Shriners Hospital), 1:1000; CD31: Santa Cruz (Dallas, TX), 1:250; GFP: Cell Signaling (Darivers, MA, USA), 1:250) overnight at 4°C. The antibodies were visualized by incubation with appropriate biotinylated antibodies (Vector Lab, Burlingame, CA, USA) followed by the color development method using the ImmPACT NovaRED Peroxidase Substrate kit (Vector Lab). The sections were counterstained with Fast Green. For immunofluorescence staining for α5 integrin, the frozen sections were incubated with the primary antibody against α5 integrin (BD BioSciences, San Jose, CA, USA) followed by incubation with the biotinylated antibodies (Vector Lab) and Texas-Red NeutrAvidin (Thermo Fisher Scientific, Waltham, MA, USA). The nuclei were stained with DAPI. Staining data were examined with a Nikon Eclipse TE400 equipped with SPOT 5.0 Advanced software (Diagnostic Instruments Inc., Sterling Heights, MI, USA), a Nikon Eclipse TE2000 microscope equipped with an Image-Pro 7.0 software (Media Cyberkinetics, Rockville, MD, USA), or Leica TCS LSI confocal macroscope system (Buffalo Grove, IL, USA). The ratio of TUNEL positive cells in sections was determined using images captured with Image-Pro 7.0 software. The number of fluorescence-labeled cells was divided by the total number of cells across all articular cartilage zones (n = 6/group).

### Histological evaluation of osteoarthritic changes

The slides of knee joints were stained with hematoxylin and eosin or Safranin O in increments of five slides and inspected by two independent researchers. The extent of changes in medial tibial plateau and medial femoral condyle of articular cartilage was evaluated using Mankin and OARSI scoring systems [22]. The extent of changes in the synovium was evaluated following the scale from a previous report [23]: 0, normal (synovial lining 1–3 cells thick); 1, mild inflammation (synovial lining 4 or 5 cells thick, increased cellularity); 2, moderate inflammation (synovial lining 6–8 cells thick and/or increase in cellularity); and 3, severe inflammation (synovial lining >9 cells thick and dense cellularity). The synovium adjacent to the meniscus toward the femur and the tibia were examined separately (4–5 sections/mouse).

### Nanoindentation

A separate group of mice were used for atomic force microscopy (AFM)-based nanoindentation, following our previously established procedures [24].

Briefly, the condyles from hind knees were dissected immediately after euthanasia by carbon dioxide, and mounted on steel AFM sample disks. Through this process, the joints were kept in sterile phosphate buffered saline (PBS) with protease inhibitors to minimize post-mortem degradation. Nanoindentation was performed on the surfaces of medial condyle cartilage using a borosilicate microspherical tip (*R* ≈ 5 μm, nominal spring constant *k* ≈ 7.4 N/m, AIO-TL tip C, NanoAndMore) and a Dimension Icon AFM (BrukerNano). On each condyle, at least 10–15 different locations were tested up to an indentation depth of ~ 1 μm at an approximately 10 μm/s indentation rate. The effective modulus, *E*_*ind*_, was calculated by fitting the loading portion of each indentation force-depth curve to the elastic Hertz model via least squares linear regression, *F* = 4*E*_ind_*R*^1/2^*D*^3/2^/[3(1 – *ν*^2^)], where *R* is the tip radius, and *ν* is Poisson’s ratio (~0.1 for cartilage) [25].

### Statistics

Student’s t-tests or two-way factorial ANOVA followed by Bonferroni post-hoc multiple comparison tests were used to identify the differences. The threshold for significance for all tests was set as p<0.05.

## Results

### 1. Ablation of α5 integrin in synovial joints in mice

**Fig 1:**
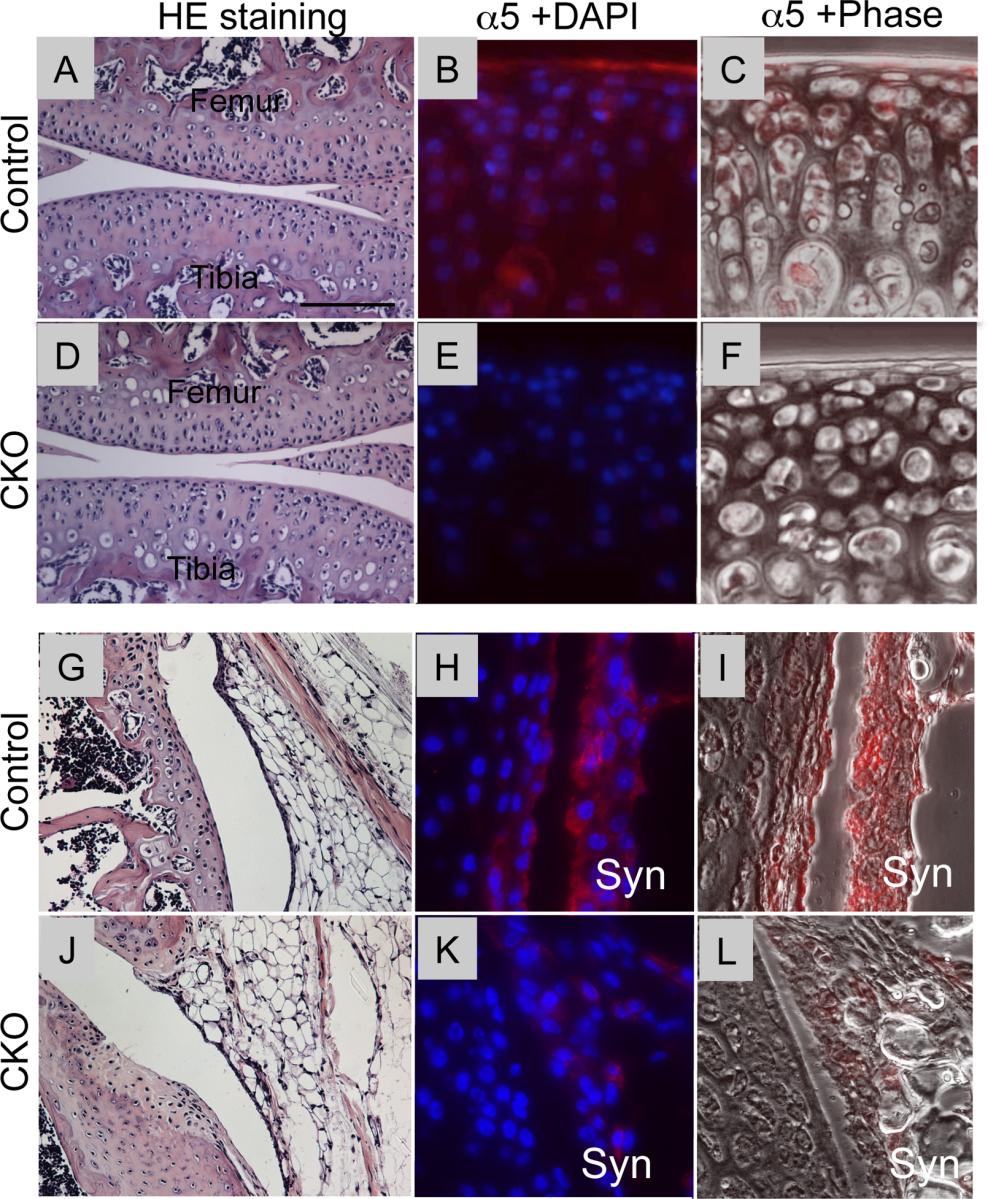
Histology and expression of α5 integrin in synovial joints in control and α5 CKO mice. The knee joints were harvested from control (A-C and G-I) and α5 CKO (D-F and J-L) at 4 weeks of age and subjected to histological inspection (A, D, G and J) and immunofluorescence staining of α5 integrin (B, E, H and K). A, D, G and J, the images of articular cartilage (A and D) and synovium (G and J) stained with hematoxylin-eosin. B, E, H and K, Superimposed images of α5 integrin staining (red) with DAPI nuclear staining (blue). C, F, I and L, Superimposed images of α5 integrin staining with the phase contrast images. Bar, 250 μm for A, D, G and J; 40 μm for B, C, E, F, H, I, K and L.

The *GDF5Cre;* α*5fl*/- mice were born normally and had an overall appearance similar to the control mice. Histological analysis of the knee joints of the GDF5Cre; α5fl/- mice did not show significant abnormalities in the structure of synovial joints including articular cartilage (Fig 1A vs 1D) and synovium (Fig 1G vs 1J) compared to the control mice. In the control mice, expression of α5 integrin was strongly expressed in articular cartilage at P0 (S1 Fig) and gradually decreased, but remained in the articular surface (Fig 1B and S1 Fig) at 4 weeks of age. α5 expression of the synovium was very strong (Fig 1H). In contrast, the α5 CKO mouse did not show positive staining for α5 integrin in articular cartilage (Fig 1E and S1 Fig) and presented much weaker staining in synovium (Fig 1K) while the perichondrium adjacent to growth plate showed α5 staining similar to the control mice (S1 Fig).

### 2. Analyses of osteoarthritic changes in the α5 CKO articular cartilage

**Fig 2:**
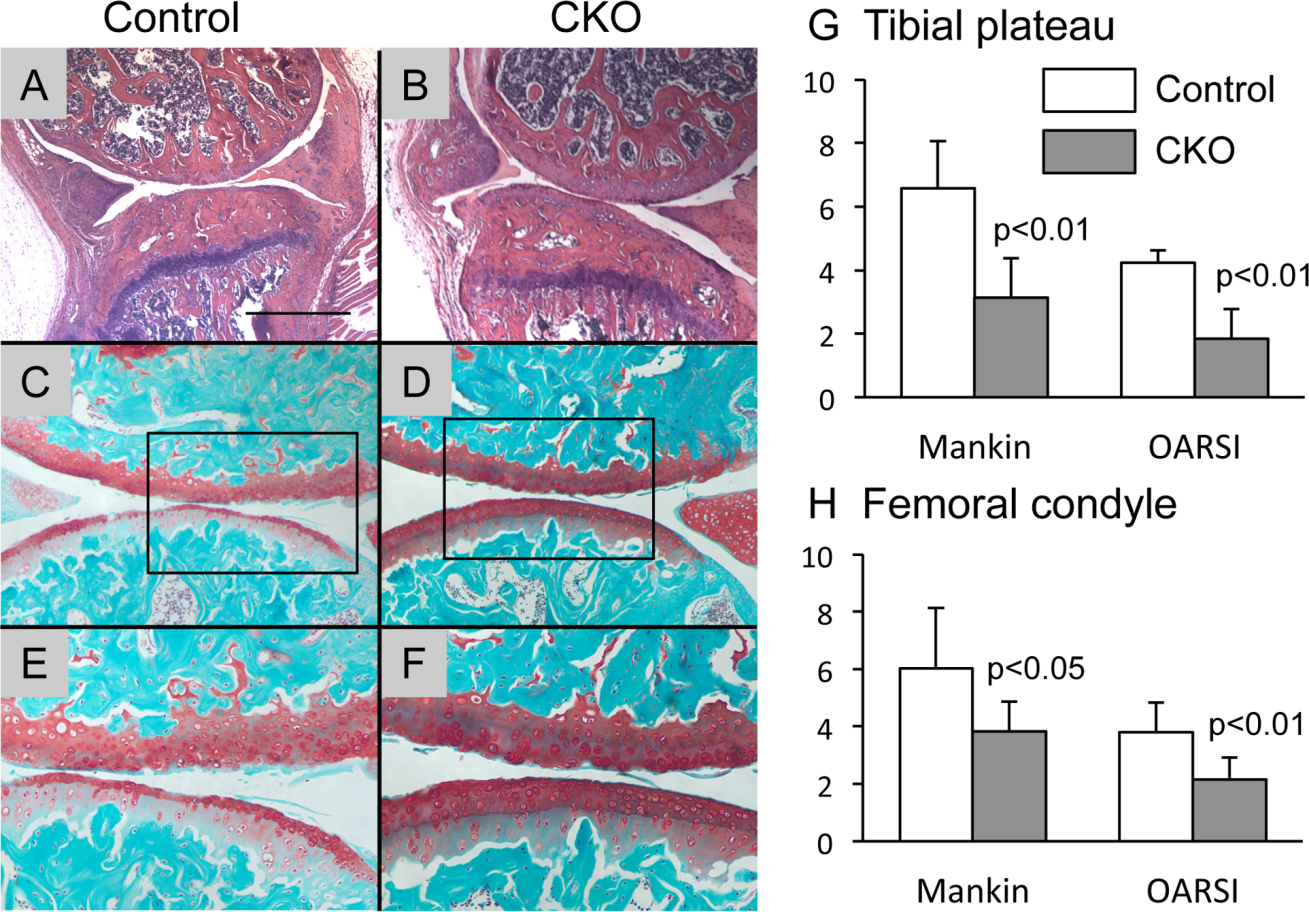
Osteoarthritic changes in control and α5 CKO mice. The knee joints were harvested from control (A, C and E) and α5 CKO (B, D and F) 4 weeks after OA surgery and subjected to histological inspection. A-F, representative images of the medial femoral condyle and the medial tibial plateau stained with hematoxylin-eosin (A and B) and Safranin O (C-F). E and F are magnified images of the box of C and D, respectively. Bar, 625 μm for A and B; 250 μm for C and D; 125 μm for E and F. G and H, Mankin and OARSI scores of the medial tibial plateau (G) and the medial femoral condyle (H). The graphs represent average and SD, n = 9/group.

Next we compared the osteoarthritic changes between the α5 CKO and the control mice after OA surgery. Interestingly, we observed significantly milder histological osteoarthritic changes in the tibial medial plateau and femoral medial condyle of the α5 CKO mice (Fig 2B, 2D and 2F) than those in the control mice (Fig 2A, 2C and 2E). Both matrix loss and structural changes were less evident in the α5 CKO articular cartilage compared to the control articular cartilage. Histological evaluation by Mankin and OARSI score methods demonstrated that the α5 CKO articular cartilage in the OA surgery side showed significantly lower values than the control articular cartilage (Fig 2G and 2H).

**Fig 3:**
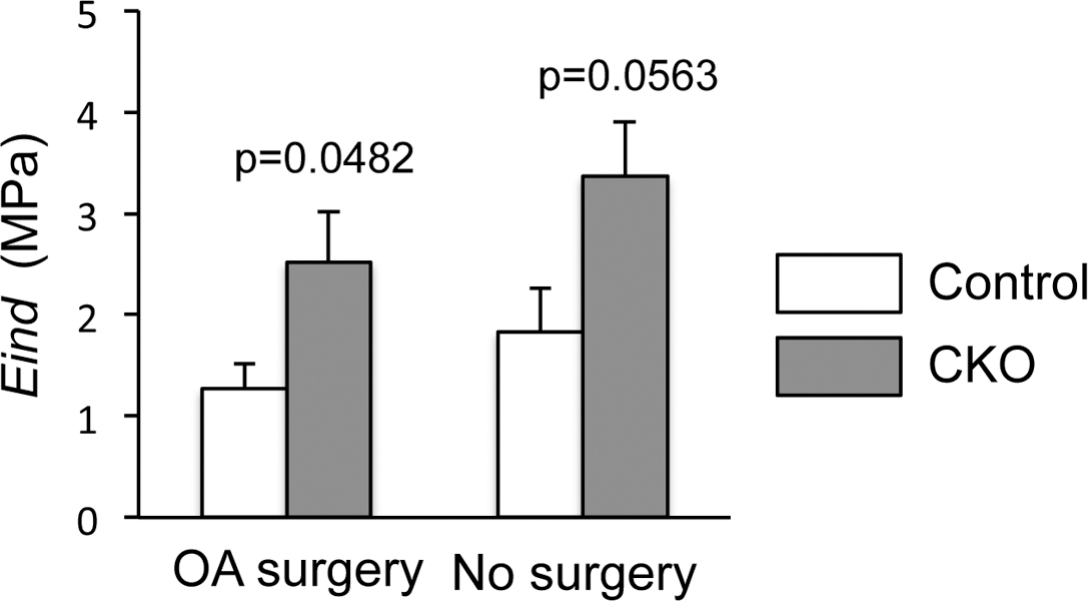
Biomechanical properties in the articular cartilage in control and α5 CKO mice. The knee joints were harvested from control and α5 CKO mice 4 weeks after OA surgery (OA surgery) and without surgery (No surgery). The femoral condyle was subjected to AFM-based nanoindentation assessment. The graph represents average and SE, n = 5/group. *Eind*, Effective indentation module calculated from Hertz model.

To examine whether milder histological changes in the α5 CKO articular cartilage was related to the changes in cartilage biomechanical properties, we performed AFM-nanoindentation assessment on the femoral condyles with or without surgery. The α5 CKO articular cartilage with OA surgery showed a significantly higher effective indentation modulus *(Eind)* than the control (Fig 3, p = 0.0482). In addition, the average of the *Eind* of the α5 CKO articular cartilage without surgery was found to be marginally higher than the control (*p* = 0.0563, Fig 3).

**Fig 4:**
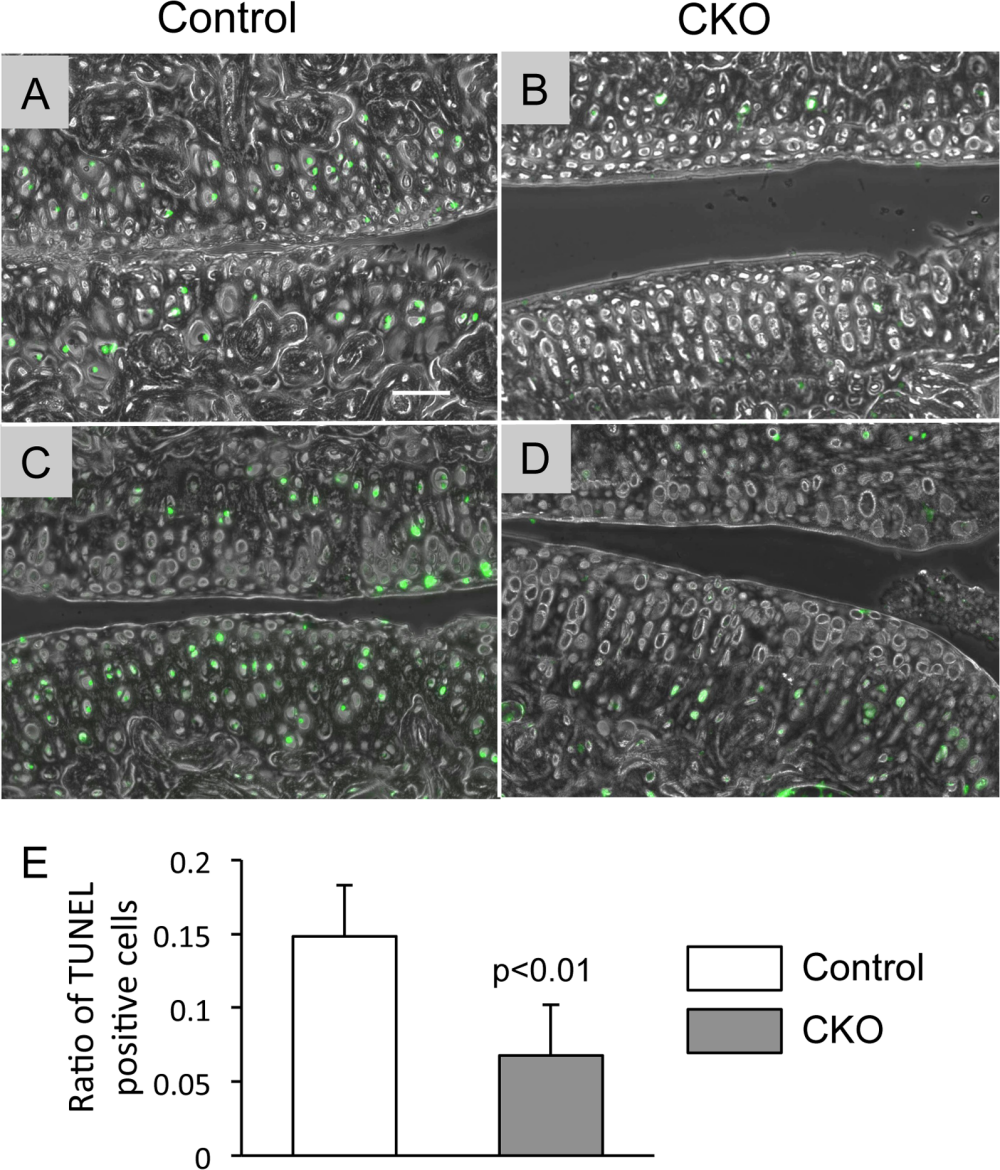
Apoptotic cells in control and α5 CKO articular cartilage after OA surgery. The knee joints were harvested from control (A and C) and α5 CKO (B and D) 4 weeks after OA surgery. The sagittal sections were subjected to TUNEL staining. The fluorescence images (green) were superimposed with the corresponding phase images. Bar, 40 μm. E, The ratio of the TUNEL-positive cells to total cells was calculated. The graphs represent average and SD (n = 6/group).

To gain more insights into understanding pathological changes in articular cartilage in the α5 CKO mice, we examined typical molecular changes associated with OA. The α5 CKO group (Fig 4A and 4C) contained a smaller number of TUNEL-positive cells than the control group (Fig 4B and 4D) (S2 Fig, TUNEL). Semi-quantitative analysis for number of TUNEL positive cells confirmed fewer TUNEL-positive cells in the a5 CKO group than the control group (Fig 4E). Expression of collagen 10, a hypertrophic marker, was found in the eroded deep zone of the control articular cartilage (S2E and S2F Fig, arrows). The α5 CKO articular cartilage had also collagen 10-positive cells throughout the entire zones (S2G and S2H Fig). The MMP13-positive cells were found in both α5 CKO (S2K and S2L Fig, arrows) and control (S2I and S2J Fig, arrows) articular cartilage. The VDIPEN staining revealed that the neo-products of aggrecan cleaved by MMPs were present in both the control and α5 CKO articular cartilage (S2 Fig, VDIPEN, arrows).

### 3. Analysis of synovial changes in the α5 CKO mice

**Fig 5:**
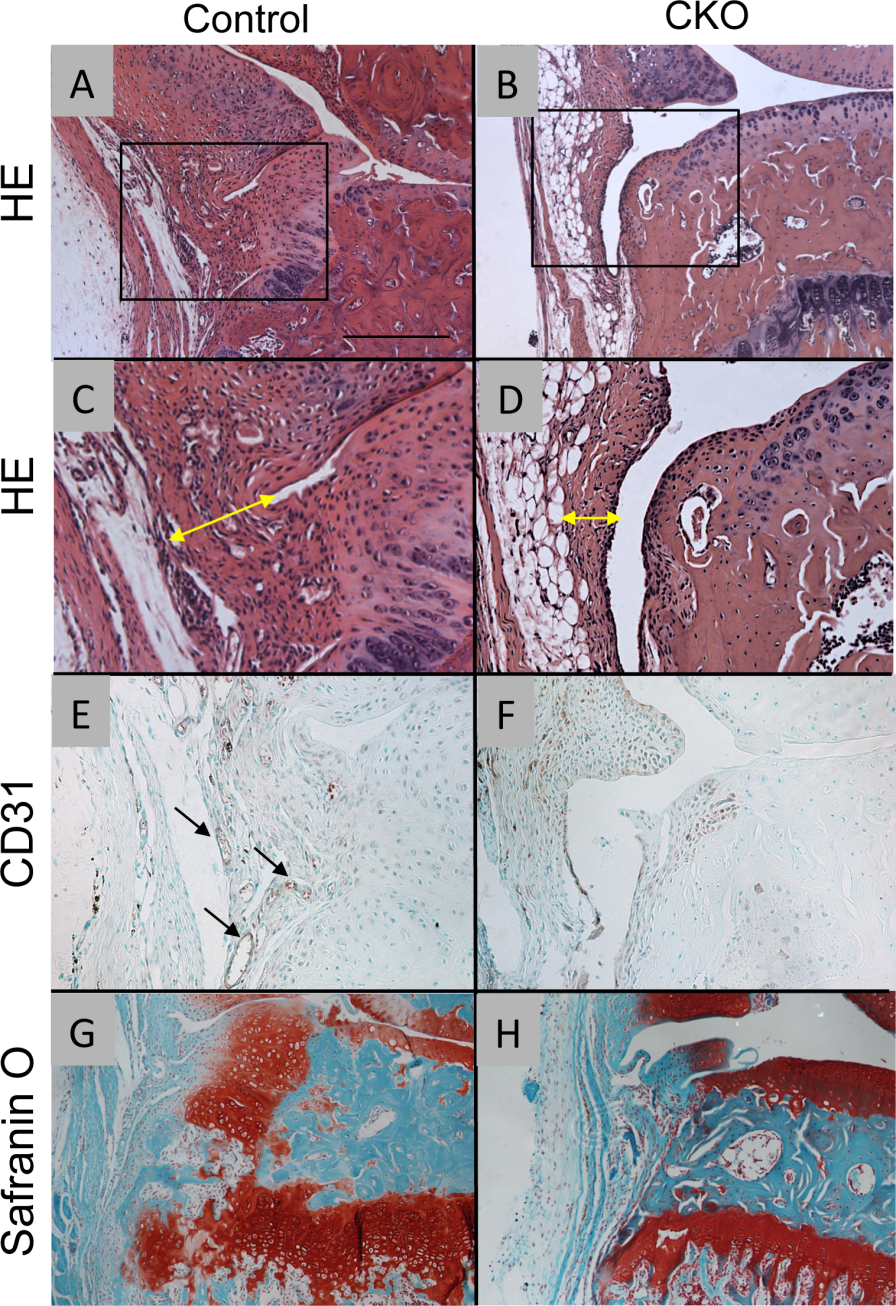
Synovial changes in control and α5 CKO mice after OA surgery. The knee joints were harvested from control (A, C, E and G) and α5 CKO (B, D, F and H) 4 weeks after OA surgery. The sagittal sections were subjected to hematoxylin-eosin staining (HE, A-D), CD31 immunostaining (E and F) and Safranin O staining (G and H). C and D are magnified images of the box of A and B, respectively. Double headed arrows indicate thickening of synovium (C and D). Arrows indicate CD31-positive vessels (E). Bar, 250 μm for A, B, G and H; 125 μm for C-F.

**Fig 6:**
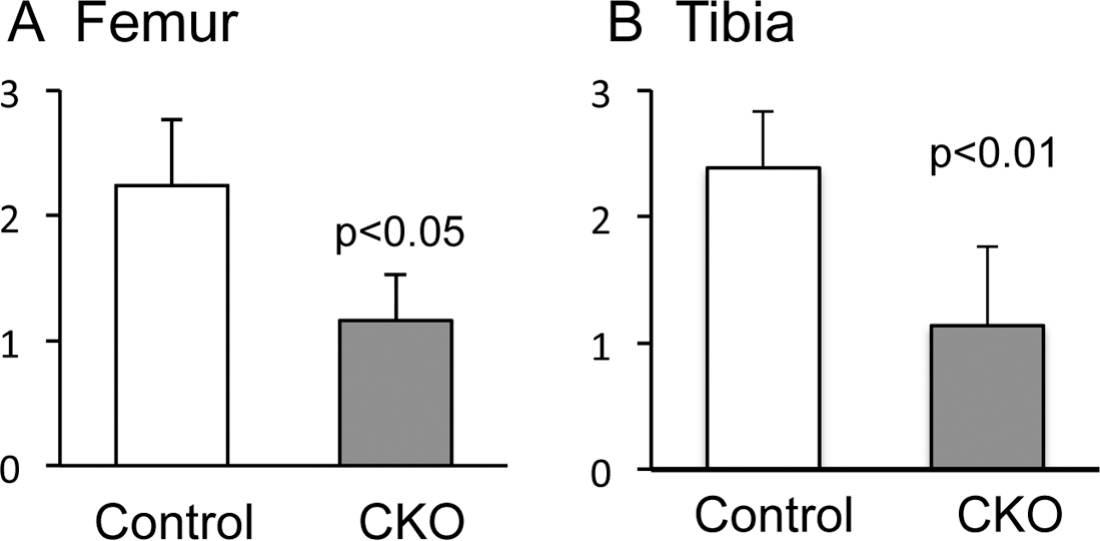
Scores of synovial changes in control and α5 CKO mice after OA surgery. Synovial changes at the medial sites beside the femur (A) and the tibia (B) were evaluated in the control and α5 CKO mice 4 weeks after OA surgery. The graphs represent average and SD, n = 9/group.

The knee joints of the control mice showed more evident hyperplasia of synovium (Fig 5A and 5C) compared to the α5 CKO mice (Fig 5B and 5D). The control synovial lining tissue had multi-layer of cells with high cellularity (Fig 5C, double headed arrow) containing CD31-positive vessels (Fig 5E, arrows). The difference in synovial change between the control and the α5 CKO group was confirmed by semi-quantitative analysis (Fig 6). Moreover, the margin of medial tibia in control mice contained ectopic cartilage (Fig 5G), while that of the α5 CKO mice did not (Fig 5H).

### 4. α5 integrin expression in the OA joints

**Fig 7:**
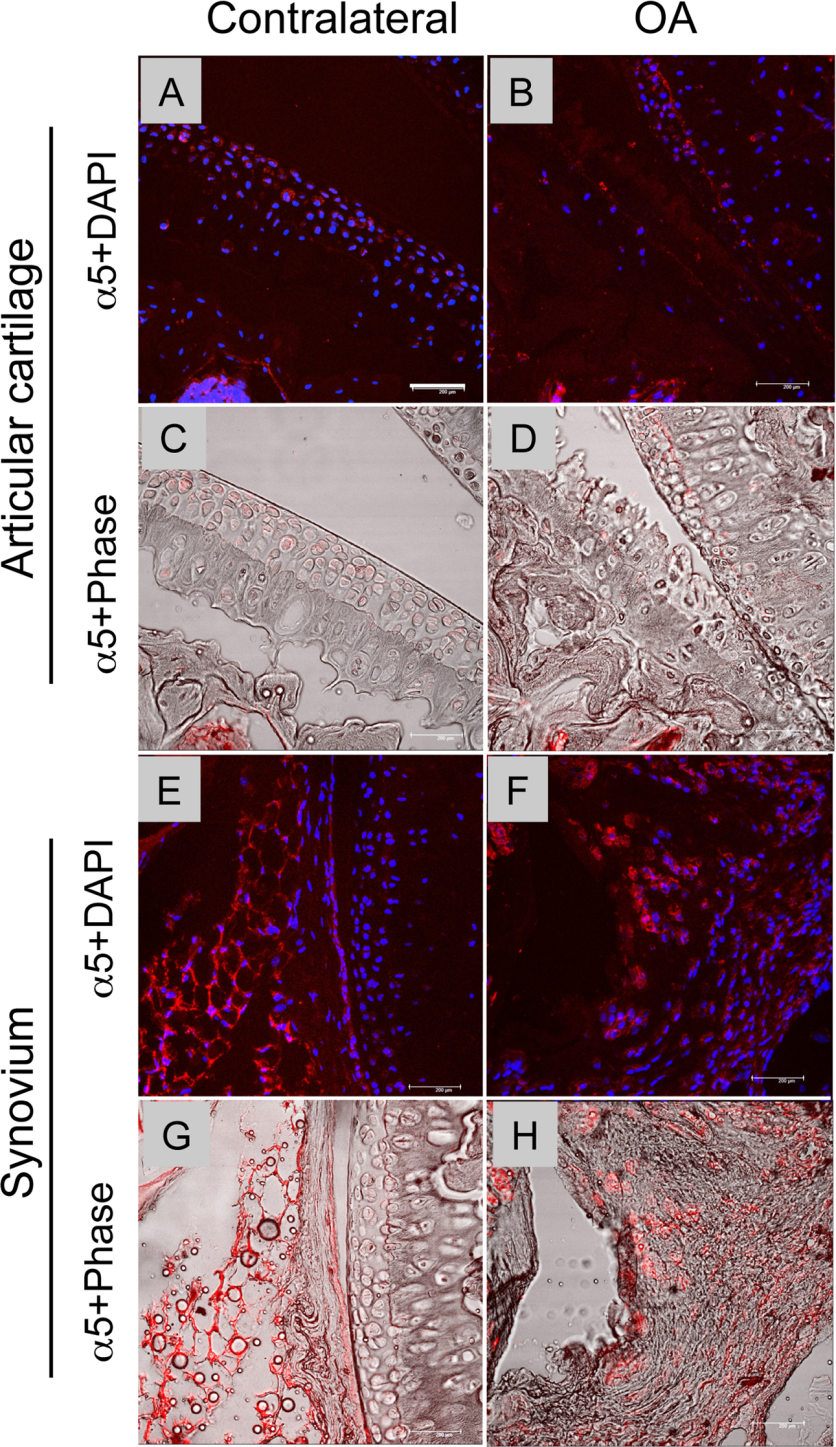
Distribution of α5 integrin in the OA and healthy knee joints. The knee joints were harvested from the surgery site (B, D, F and H) and the contralateral site (A, C, E and G) 4 weeks after OA surgery. A, B, E and F, Superimposed images of α5 integrin staining (red) with DAPI nuclear staining (blue). C, D, G and H, Superimposed images of α5 integrin staining (red) with the phase contrast images. Bar, 40 μm.

We examined changes in α65 integrin expression after OA surgery. The staining of α5 integrin was weaker in the OA articular cartilage than that in the normal articular cartilage (Fig 7A vs 7B). In contrast, α5 integrin was found throughout the hyperplastic lesion of the synovium (Fig 7F). These findings suggest that ablation of α5 integrin in the synovium results in reduction in synovial changes. To examine how Gdf5-lineage cells behave in OA, the *Gdf5;Rosa-GFP* reporter mice received OA surgery. Four weeks after surgery we examined which cells express GFP to monitor the distribution of GDF5-lineage cells in the knee joints. As previously reported, the entire articular cartilage is composed of GFP-positive cells (S3 Fig). Interestingly, GFP expression was weaker at the surgery site than at the contralateral site (S3 Fig), suggesting that the basal level of protein expression may decrease in the OA articular cartilage. GFP expression was found in the synovial cells that represent hyperplasia (S3 Fig) at the surgery site, indicating the synovial changes involve Gdf5-lineage cells.

## Discussion

Results in this study demonstrated that while α5 integrin may not be essential for synovial joint development and structural maturation, it can play an important role in osteoarthritic changes in articular cartilage and synovial tissue. Osteoarthritis is the most common joint disease and causes clinical symptoms, including pain and stiffness. The results from this study provide important insights into the mechanism of osteoarthritis and would pave the way toward the development of new and effective therapeutic drugs to prevent or ameliorate trauma-induced OA.

### The role of α5 integrin in chondrocytes

We observed here that ablation of α5 integrin reduced matrix loss and structural changes in articular cartilage after OA surgery. The α5 CKO articular cartilage showed smaller values of the modified Mankin score and OARSI score (Fig 2H and 2G). Furthermore, the α5 CKO articular cartilage also had a higher nanoindentation modulus (Fig 3), indicating a better load bearing function than wild-type cartilage. At the cellular level, the number of apoptotic cells was significantly smaller in the α5 CKO than that in the control (Fig 4). The finding suggests that α5 integrin may regulate osteoarthritic responses through mediating the apoptotic signal. Pulai et al. [14] have demonstrated that incubation with the α5β1 antibodies induces cell death of articular chondrocytes in suspension culture and concluded that α5β1 integrin provides matrix survival signals. In contrast, we observed a decrease in apoptotic cells in the α5 CKO articular cartilage, suggesting that α5 integrin participates in cell apoptosis. The discrepancy can be accounted for through the following explanations. First, previous findings are based on *in vitro* culture experiments while the results in this study were from *in vivo* experiments. Articular chondrocytes behave differently *in vitro* than *in vivo*. Furthermore, the articular chondrocytes are influenced *in vivo* by many factors that are locally provided in other joint tissues and systemically circulated. Second, most previous studies have used functional antibodies against α5β1 integrin to inactivate α5β1 integrin while our study involves ablation of α5 integrin alone. Functional antibodies might induce other confounding intracellular signaling in addition to the deletion of α5-mediated signal.

Previous reports have demonstrated that α5 β1 integrin mediates matrix degradation induced by fibronectin fragments in cultured chondrocytes [15]. Furthermore, the expression of inflammatory-related molecules, IL-1β, IL-6, IL-8, PGE2 and nitrite (NO) were stimulated in articular chondrocytes when they are treated with the functional antibodies of α5β1 [17]. The phenotype of the α5 CKO mice observed in this study is likely consistent with these findings. The levels of fibronectin [26, 27] and fibronectin fragments (30–200 kD) are increased in OA articular cartilage and synovial fluid [28]. Fibronectin fragments are strong stimulators of MMPs [29]. Therefore, the absence of α5 integrin may attenuate fibronectin fragment-induced matrix degradation in the α5 CKO mice. It has been demonstrated that α5 integrin expression increased in articular cartilage after partial menisectomy OA surgery in rats [30, 31] while our results showed reduction in its expression (Fig 7). A decrease in α5 integrin staining was observed particularly in the region that showed severe structural defects. Thus the reduction in α5 integrin expression seen in our model may be associated with reduction in cell metabolic activity or cell death. This assumption is consistent with our findings that articular cartilage at the surgery side contained many apoptotic cells (Fig 4) and a lower level of GFP expression in the *GDF5;Rosa-GFP* mice compared to the articular cartilage at the contralateral side (S3 Fig).

### The role of α5 integrin in synovial changes

The OA model we used in this study is a hemisectomy of medial meniscus in addition to incision of medial collateral ligament, which is a more severe condition than the DMM model that involves transection of the medial meniscotibial ligament [32]. We observed obvious synovial changes at an earlier time point than in the DMM model. The synovium and fat pad expressed α5 integrin at a higher level than articular cartilage. The Gdf5Cre system induces Cre activity in articular cartilage but also does so in other joint components, including synovium and fat pad [33]. We demonstrated that thickened synovial tissue is composed of the cells labeled with Gdf5Cre (S3 Fig) and showed strong staining for α5 integrin. Thus, deletion of α5 integrin may result in a marked impact on synovium. Indeed, synovial changes in the control mice were much more evident than in the α5 CKO mice.

Synovial changes observed in the control group include hyperplasia of synovium (Fig 5A and 5C), an increase in vessels (Fig 5E), and appearance of ectopic cartilaginous lesions (Fig 5G). In the α5 CKO group, thickening of the synovium (Fig 5B and 5D and Fig 6) and ectopic cartilage formation (Fig 5H) were to a lesser extent, indicating that α5 integrin mediates these changes directly or indirectly. α5 integrin has been demonstrated to regulate cell proliferation via cell adhesion or spreading [34, 35]. Furthermore, α5 and α v integrins contribute to embryonic vascular development and tumor angiogenesis [9, 36]. Thus, contribution of α5 integrin to hyperplasia of the synovium and vessel formation may be direct. Previous reports have shown that α5 integrin is involved in up-regulation of MMPs (MMP-1, 3 and 13) and inflammatory cytokines (IL-1α and IL-1β) in synovial cells in response to fibronectin fragments [37, 38], suggesting that α5 integrin-mediated up-regulation of MMPs and inflammatory cytokines in synovial cells may result in stimulation of loss of matrix and destruction of cartilage. Requirement of α5 integrin has been reported in growth plate chondrocytes in suspension culture [39]. Therefore, absence of α5 integrin would inhibit the growth of ectopic cartilaginous lesions. We need further investigation to elucidate how α5 integrin participates in induction of ectopic cartilage formation.

## Conclusions

In summation, our results demonstrate that ablation of α5 integrin in synovial joints reduces susceptibility to osteoarthritic changes in mice and suggest that α5 integrin is a stimulator of osteoarthritis.

## Supporting Information

S1 Fig Expression of α5 integrin in synovial joints in control and α5 CKO mice. The knee joints were harvested at P0 (A and B) or 4 weeks of age (C-F) from the C57BL/6j mice (A and B), control (C and D) and the α5 CKO (E and F) mice. The α5 staining images (red) are superimposed with DAPI nuclear staining (blue). B is the magnified image of the box in A. Bar, 40 μm.
(TIF)

S2 Fig Apoptotic cells, expression of collagen 10 and MMP13 and cleaved aggrecan products in control and α5 CKO articular cartilage after OA surgery. The knee joints were harvested from control (A, B, E, F, I, J, M and N) and α5 CKO (C, D, G, H, K, L, O and P) 4 weeks after OA surgery. The sagittal sections were subjected to TUNEL (A-D), collagen 10 (E-H), MMP13 (I-L) and VDIPEN (M-P) staining. B, D, F, H, J, L, N and P are magnified images of the box of A, C, E, G, I, K, M and O, respectively. Arrows indicate the cells or regions that represent positive immuno-reactivity to the corresponding staining. Bar, 250 μm for A, C, E, G, I, K, M and O; 125 μm for B, D, F, H, J, L, N and P.
(TIF)

S3 Fig Expression of GFP in the knee joints after OA surgery. Sagittal sections were made from the OA surgery knee joint (A and C) and the contralateral knee joint (B and D) in the Gdf5CRe;Rosa-GFP mouse and subjected to GFP immunostaining. A and B, articular cartilage. C and D, synovium adjacent to medial tibia. Bar,125 μm.
(TIF)

## References

[1] Pritzker KP, Gay S, Jimenez SA, Ostergaard K, Pelletier JP, Revell PA, et al Osteoarthritis cartilage histopathology: grading and staging. Osteoarthritis Cartilage. 2006;14: 13–29. 16242352 10.1016/j.joca.2005.07.014

[2] Goldring MB, Goldring SR. Osteoarthritis. J Cell Physiol. 2007;213: 626–634. 17786965 10.1002/jcp.21258

[3] van der Kraan PM, van den Berg WB. Osteophytes: relevance and biology. Osteoarthritis Cartilage. 2007;15: 237–244. 17204437 10.1016/j.joca.2006.11.006

[4] Lories RJ, Luyten FP. The bone-cartilage unit in osteoarthritis. Nat Rev Rheumatol. 2011;7: 43–49. 10.1038/nrrheum.2010.197 21135881

[5] Scanzello CR, Goldring SR. The role of synovitis in osteoarthritis pathogenesis. Bone. 2012;51: 249–257. 10.1016/j.bone.2012.02.012 22387238 PMC3372675

[6] Wenham CY, Conaghan PG. The role of synovitis in osteoarthritis. Ther Adv Musculoskelet Dis. 2010;2: 349–359. 10.1177/1759720X10378373 22870460 PMC3383490

[7] Hynes RO. Integrins: bidirectional, allosteric signaling machines. Cell. 2002;110: 673–687. 12297042 10.1016/s0092-8674(02)00971-6

[8] Yang JT, Rayburn H, Hynes RO. Embryonic mesodermal defects in alpha 5 integrin-deficient mice. Development. 1993;119: 1093–1105. 7508365 10.1242/dev.119.4.1093

[9] van der Flier A, Badu-Nkansah K, Whittaker CA, Crowley D, Bronson RT, Lacy-Hulbert A, et al Endothelial alpha5 and alphav integrins cooperate in remodeling of the vasculature during development. Development. 2010;137: 2439–2449. 10.1242/dev.049551 20570943 PMC2889609

[10] Turner CJ, Badu-Nkansah K, Crowley D, van der Flier A, Hynes RO. alpha5 and alphav integrins cooperate to regulate vascular smooth muscle and neural crest functions in vivo. Development. 2015;142: 797–808. 10.1242/dev.117572 25670798 PMC6514387

[11] Salter DM, Godolphin JL, Gourlay MS. Chondrocyte heterogeneity: immunohistologically defined variation of integrin expression at different sites in human fetal knees. J Histochem Cytochem. 1995;43: 447–457. 7897185 10.1177/43.4.7897185

[12] Woods VL Jr, Schreck PJ, Gesink DS, Pacheco HO, Amiel D, Akeson WH, et al Integrin expression by human articular chondrocytes. Arthritis Rheum. 1994;37: 537–544. 8147931 10.1002/art.1780370414

[13] Garciadiego-Cazares D, Rosales C, Katoh M, Chimal-Monroy J. Coordination of chondrocyte differentiation and joint formation by alpha5beta1 integrin in the developing appendicular skeleton. Development. 2004;131: 4735–4742. 15329344 10.1242/dev.01345

[14] Pulai JI, Del Carlo M Jr, Loeser RF. The alpha5beta1 integrin provides matrix survival signals for normal and osteoarthritic human articular chondrocytes in vitro. Arthritis Rheum. 2002;46: 1528–1535. 12115183 10.1002/art.10334

[15] Forsyth CB, Pulai J, Loeser RF. Fibronectin fragments and blocking antibodies to alpha2beta1 and alpha5beta1 integrins stimulate mitogen-activated protein kinase signaling and increase collagenase 3 (matrix metalloproteinase 13) production by human articular chondrocytes. Arthritis Rheum. 2002;46: 2368–2376. 12355484 10.1002/art.10502

[16] Homandberg GA, Costa V, Ummadi V, Pichika R. Antisense oligonucleotides to the integrin receptor subunit alpha(5) decrease fibronectin fragment mediated cartilage chondrolysis. Osteoarthritis Cartilage. 2002;10: 381–393. 12027539 10.1053/joca.2002.0524

[17] Attur MG, Dave MN, Clancy RM, Patel IR, Abramson SB, Amin AR. Functional genomic analysis in arthritis-affected cartilage: yin-yang regulation of inflammatory mediators by alpha 5 beta 1 and alpha V beta 3 integrins. J Immunol. 2000;164: 2684–2691. 10679109 10.4049/jimmunol.164.5.2684

[18] Tanaka N, Ikeda Y, Yamaguchi T, Furukawa H, Mitomi H, Nakagawa T, et al alpha5beta1 integrin induces the expression of noncartilaginous procollagen gene expression in articular chondrocytes cultured in monolayers. Arthritis Res Ther. 2013;15: R127 10.1186/ar4307 24286194 PMC3978676

[19] Rountree RB, Schoor M, Chen H, Marks ME, Harley V, Mishina Y, et al BMP receptor signaling is required for postnatal maintenance of articular cartilage. PLoS Biol. 2004;2: e355 15492776 10.1371/journal.pbio.0020355 PMC523229

[20] Koyama E, Shibukawa Y, Nagayama M, Sugito H, Young B, Yuasa T, et al A distinct cohort of progenitor cells participates in synovial joint and articular cartilage formation during mouse limb skeletogenesis. Dev Biol. 2008;316: 62–73. 10.1016/j.ydbio.2008.01.012 18295755 PMC2373417

[21] Ohta Y, Okabe T, Larmour C, Di Rocco A, Maijenburg MW, Phillips A, et al Articular cartilage endurance and resistance to osteoarthritic changes require transcription factor Erg. Arthritis Rheumatol. 2015;67: 2679–2690. 10.1002/art.39243 26097038 PMC5568074

[22] Glasson SS, Chambers MG, Van Den Berg WB, Little CB. The OARSI histopathology initiative—recommendations for histological assessments of osteoarthritis in the mouse. Osteoarthritis Cartilage. 2010;18 Suppl 3: S17–23. 10.1016/j.joca.2010.05.025 20864019

[23] Haywood L, McWilliams DF, Pearson CI, Gill SE, Ganesan A, Wilson D, et al Inflammation and angiogenesis in osteoarthritis. Arthritis Rheum. 2003;48: 2173–2177. 12905470 10.1002/art.11094

[24] Batista MA, Nia HT, Onnerfjord P, Cox KA, Ortiz C, Grodzinsky AJ, et al Nanomechanical phenotype of chondroadherin-null murine articular cartilage. Matrix Biol. 2014;38: 84–90. 10.1016/j.matbio.2014.05.008 24892719 PMC6698058

[25] Buschmann MD, Kim Y-J, Wong M, Frank E, Hunziker EB, Grodzinsky AJ. Stimulation of aggrecan synthesis in cartilage explants by cyclic loading is localized to regions of high interstitial fluid flow. Archives of Biochemistry & Biophysics. 1999;366: 1–7. 10.1006/abbi.1999.1197 10334856

[26] Brown RA, Jones KL. The synthesis and accumulation of fibronectin by human articular cartilage. J Rheumatol. 1990;17: 65–72. 2313677

[27] Lust G, Burton-Wurster N, Leipold H. Fibronectin as a marker for osteoarthritis. J Rheumatol. 1987;14 Spec No: 28–29. 3625673

[28] Homandberg GA, Wen C, Hui F. Cartilage damaging activities of fibronectin fragments derived from cartilage and synovial fluid. Osteoarthritis Cartilage. 1998;6: 231–244. 9876392 10.1053/joca.1998.0116

[29] Homandberg GA, Meyers R, Xie DL. Fibronectin fragments cause chondrolysis of bovine articular cartilage slices in culture. J Biol Chem. 1992;267: 3597–3604. 1740411

[30] Almonte-Becerril M, Costell M, Kouri JB. Changes in the integrins expression are related with the osteoarthritis severity in an experimental animal model in rats. J Orthop Res. 2014;32: 1161–1166. 10.1002/jor.22649 24839051

[31] Garciadiego-Cazares D, Aguirre-Sanchez HI, Abarca-Buis RF, Kouri JB, Velasquillo C, Ibarra C. Regulation of alpha5 and alphaV Integrin Expression by GDF-5 and BMP-7 in Chondrocyte Differentiation and Osteoarthritis. PLoS ONE. 2015;10: e0127166 10.1371/journal.pone.0127166 26010756 PMC4443976

[32] Fang H, Beier F. Mouse models of osteoarthritis: modelling risk factors and assessing outcomes. Nat Rev Rheumatol. 2014;10: 413–421. 10.1038/nrrheum.2014.46 24662645

[33] Candela ME, Yasuhara R, Iwamoto M, Enomoto-Iwamoto M. Resident mesenchymal progenitors of articular cartilage. Matrix Biol. 2014;39: 44–49. 10.1016/j.matbio.2014.08.015 25179676 PMC4435713

[34] Zhou Q, Pardo A, Konigshoff M, Eickelberg O, Budinger GR, Thavarajah K, et al Role of von Hippel-Lindau protein in fibroblast proliferation and fibrosis. Faseb J. 2011;25: 3032–3044. 10.1096/fj.10-177824 21642472 PMC3157679

[35] Davey G, Buzzai M, Assoian RK. Reduced expression of (alpha)5(beta)1 integrin prevents spreading-dependent cell proliferation. J Cell Sci. 1999;112 (Pt 24): 4663–4672. 10574714 10.1242/jcs.112.24.4663

[36] Murphy PA, Begum S, Hynes RO. Tumor angiogenesis in the absence of fibronectin or its cognate integrin receptors. PLoS ONE. 2015;10: e0120872 10.1371/journal.pone.0120872 25807551 PMC4373772

[37] Yasuda T, Shimizu M, Nakagawa T, Julovi SM, Nakamura T. Matrix metalloproteinase production by COOH-terminal heparin-binding fibronectin fragment in rheumatoid synovial cells. Lab Invest. 2003;83: 153–162. 12594231 10.1097/01.lab.0000056999.08437.b2

[38] Saito S, Yamaji N, Yasunaga N, Saito T, Matsumoto S, Katoh M, et al The fibronectin extra domain A activates matrix metalloproteinase gene expression by an interleukin-1-dependent mechanism. J Biol Chem. 1999;274: 30756–30763. 10521465 10.1074/jbc.274.43.30756

[39] Enomoto-Iwamoto M, Iwamoto M, Nakashima K, Mukudai Y, Boettiger D, Pacifici M, et al Involvement of alpha5beta1 integrin in matrix interactions and proliferation of chondrocytes. J Bone Miner Res. 1997;12: 1124–1132. 9200013 10.1359/jbmr.1997.12.7.1124

