# Supplementary figures and images for: Alpha 5 Integrin Mediates Osteoarthritic Changes in Mouse Knee Joints
Source: PLoS One. 2016 Jun 9;11(6):e0156783. doi: 10.1371/journal.pone.0156783 (PMC4900574; doi:10.1371/journal.pone.0156783)

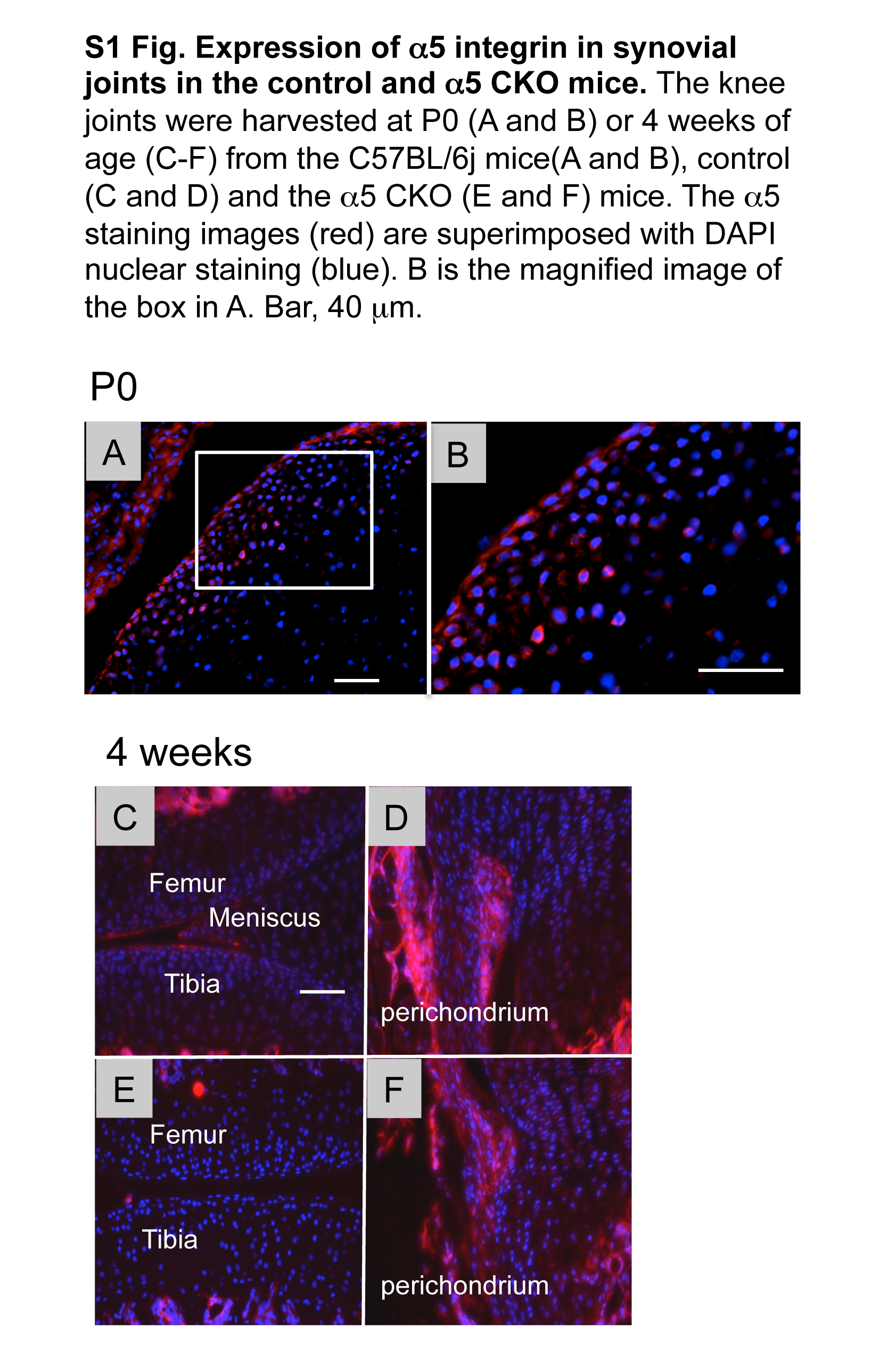

Supplement: S1 Fig — The knee joints were harvested at P0 (A and B) or 4 weeks of age (C-F) from the C57BL/6j mice (A and B), control (C and D) and the α5 CKO (E and F) mice. The α5 staining images (red) are superimposed with DAPI nuclear staining (blue). B is the magnified image of the box in A. Bar, 40 μm. (TIF) [file pone.0156783.s001.tif]

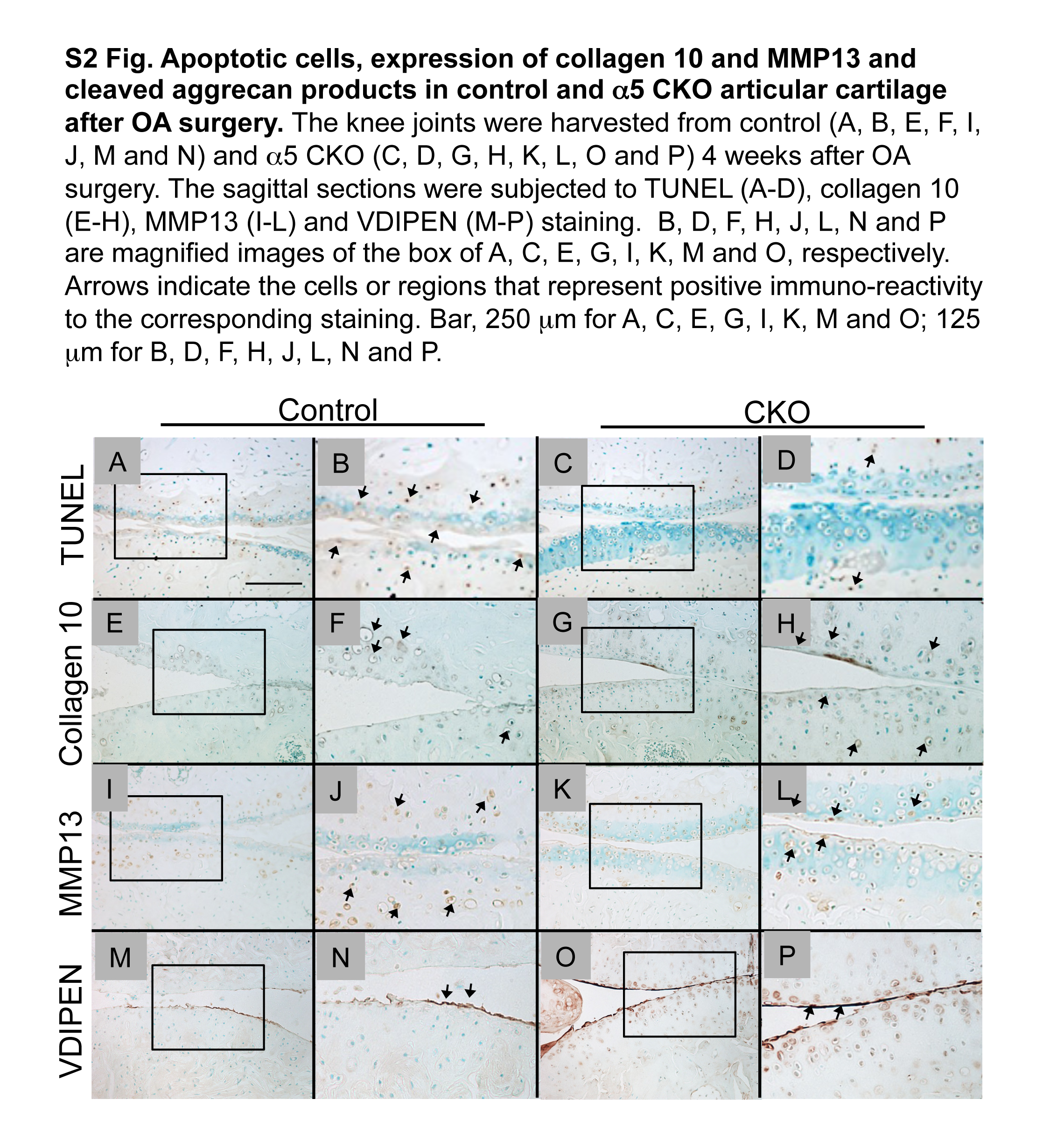

Supplement: S2 Fig — The knee joints were harvested from control (A, B, E, F, I, J, M and N) and α5 CKO (C, D, G, H, K, L, O and P) 4 weeks after OA surgery. The sagittal sections were subjected to TUNEL (A-D), collagen 10 (E-H), MMP13 (I-L) and VDIPEN (M-P) staining. B, D, F, H, J, L, N and P are magnified images of the box of A, C, E, G, I, K, M and O, respectively. Arrows indicate the cells or regions that represent positive immuno-reactivity to the corresponding staining. Bar, 250 μm for A, C, E, G, I, K, M and O; 125 μm for B, D, F, H, J, L, N and P. (TIF) [file pone.0156783.s002.tif]

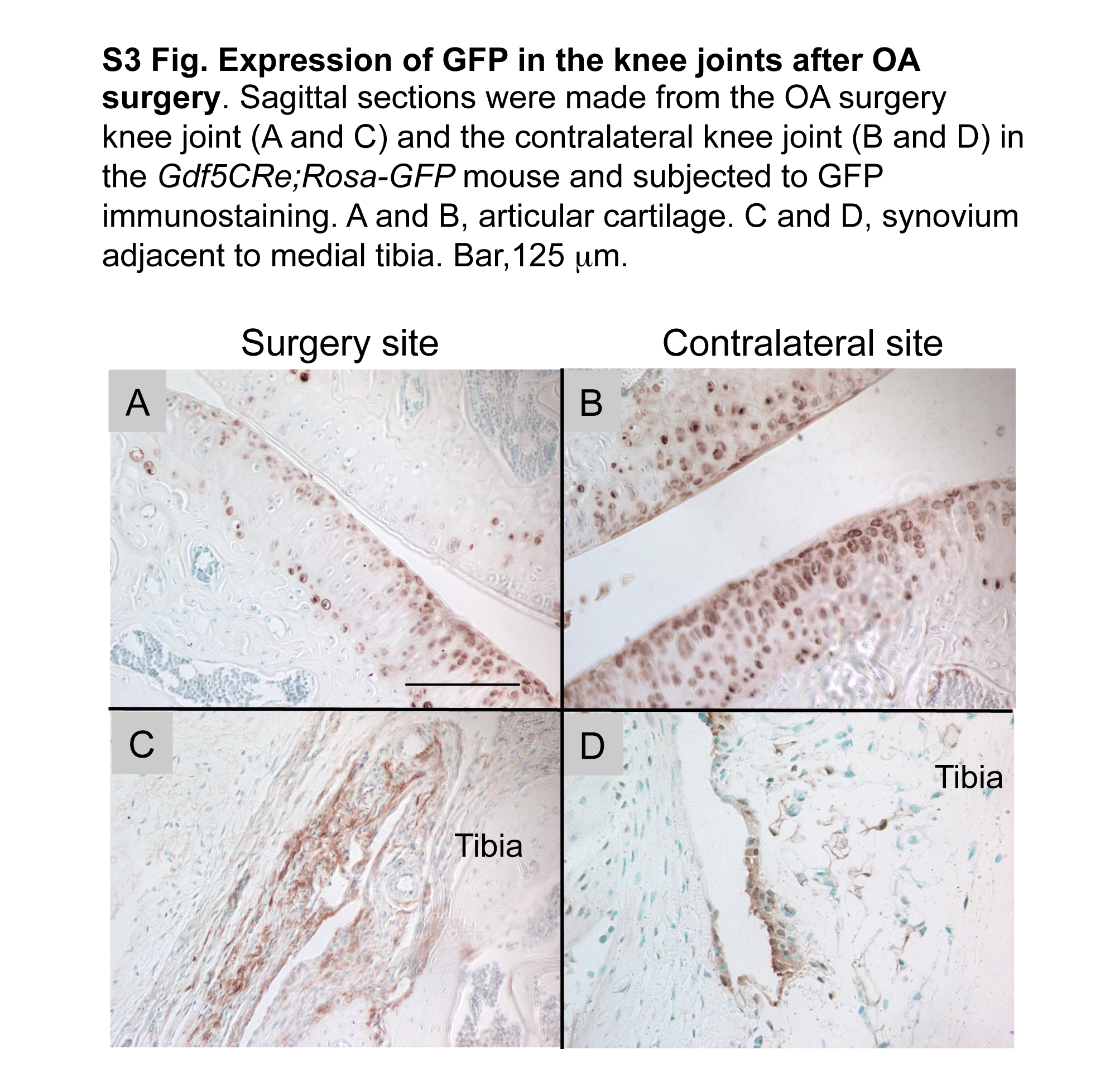

Supplement: S3 Fig — Sagittal sections were made from the OA surgery knee joint (A and C) and the contralateral knee joint (B and D) in the Gdf5CRe;Rosa-GFP mouse and subjected to GFP immunostaining. A and B, articular cartilage. C and D, synovium adjacent to medial tibia. Bar,125 μm. (TIF) [file pone.0156783.s003.tif]
